# HIV, Tuberculosis, and Food Insecurity in Africa—A Syndemics-Based Scoping Review

**DOI:** 10.3390/ijerph19031101

**Published:** 2022-01-19

**Authors:** Temitope Ojo, Christina Ruan, Tania Hameed, Carly Malburg, Sukruthi Thunga, Jaimie Smith, Dorice Vieira, Anya Snyder, Siphra Jane Tampubolon, Joyce Gyamfi, Nessa Ryan, Sahnah Lim, Michele Santacatterina, Emmanuel Peprah

**Affiliations:** 1 Implementing Sustainable Evidence-Based Interventions through Engagement (ISEE) Lab, Global Health Program, Department of Social and Behavioral Sciences, School of Global Public Health, New York University (NYU), 708 Broadway, 4th Floor, New York, NY 10003, USA; cr3041@nyu.edu (C.R.); tah10011@nyu.edu (T.H.); cm5467@nyu.edu (C.M.); st4181@nyu.edu (S.T.); js12021@nyu.edu (J.S.); dorice.vieira@nyulangone.org (D.V.); as13565@nyu.edu (A.S.); siphra.tampubolon@undp.org (S.J.T.); gyamfj01@nyu.edu (J.G.); ryann01@nyu.edu (N.R.); ep91@nyu.edu (E.P.); 2 NYU Health Sciences Library, 550 First Avenue, New York, NY 10016, USA; 3 Section for Health Equity, Department of Population Health, NYU Langone Health, 180 Madison Avenue, New York, NY 10016, USA; Sahnah.Lim@nyulangone.org; 4 Division of Biostatistics, Department of Population Health, NYU Langone Health, 180 Madison Avenue, New York, NY 10016, USA; michele.santacatterina@nyulangone.org

**Keywords:** food insecurity, HIV/AIDs, tuberculosis, syndemics, Africa

## Abstract

The double burden of HIV/AIDS and tuberculosis (TB), coupled with endemic and problematic food insecurity in Africa, can interact to negatively impact health outcomes, creating a syndemic. For people living with HIV/AIDS (PWH), food insecurity is a significant risk factor for acquiring TB due to the strong nutritional influences and co-occurring contextual barriers. We aim to synthesize evidence on the syndemic relationship between HIV/AIDS and TB co-infection and food insecurity in Africa. We conducted a scoping review of studies in Africa that included co-infected adults and children, with evidence of food insecurity, characterized by insufficient to lack of access to macronutrients. We sourced information from major public health databases. Qualitative, narrative analysis was used to synthesize the data. Of 1072 articles screened, 18 articles discussed the syndemic effect of HIV/AIDS and TB co-infection and food insecurity. Reporting of food insecurity was inconsistent, however, five studies estimated it using a validated scale. Food insecure co-infected adults had an average BMI of 16.5–18.5 kg/m^2^. Negative outcomes include death (*n* = 6 studies), depression (*n* = 1 study), treatment non-adherence, weight loss, wasting, opportunistic infections, TB-related lung diseases, lethargy. Food insecurity was a precursor to co-infection, especially with the onset/increased incidence of TB in PWH. Economic, social, and facility-level factors influenced the negative impact of food insecurity on the health of co-infected individuals. Nutritional support, economic relief, and psychosocial support minimized the harmful effects of food insecurity in HIV–TB populations. Interventions that tackle one or more components of a syndemic interaction can have beneficial effects on health outcomes and experiences of PWH with TB in Africa.

## 1. Introduction

Joint United Nations Programme on HIV/AIDs (UNAIDS) data estimate that 25.5 million people are living with HIV/AIDs (PWH) in Africa, which accounts for 68% of the global population living with HIV/AIDS [1]. Although the use of highly active antiretroviral therapy (HAART) has significantly prolonged the life expectancy of PWH and improved health outcomes, Africa continues to experience a high incidence of HIV/AIDS with two-thirds of all new global cases occurring on the continent [2]. The continent has yet to reach the 90-90-90 global target of increasing awareness of HIV status, accessing treatment, and suppressing viral load. The 90-90-90 target states that by 2020, 90% of people living with HIV should be diagnosed, 90% of those diagnosed treated, and 90% of those treated virally suppressed [3]. Currently, only 47–85% of PWH know their status globally, 29–67% of those with HIV have access to antiretroviral therapy (ART), and 22–57% have suppressed viral load [3]. Although gains throughout the past couple of years have been made in getting Africa closer to the 90-90-90 targets, there remain underlying contextual barriers to achieving optimal HIV prevention and management.

One significant contextual barrier is food insecurity. Successful HIV/AIDS management relies on optimal nutritional status as a core component in a successful treatment regime, as good nutrition supports the positive immune response to antiretroviral therapy (ART) [4]. HIV-related weight loss and wasting, due to low energy intake and increased energy demands from HIV infection [5,6], may be complicated within a context of food insecurity and low income, and become a risk factor for HIV progression and mortality [7,8]. Food insecurity is the “lack of regular access to safe and nutritious food for normal growth and development and an active and healthy life, due to unavailability of food and/or lack of resources to obtain food” [9]. Food insecurity can be experienced at different levels of severity—mild, moderate, and severe. The severity of food insecurity progresses cumulatively by uncertainty in accessing food, insufficient resources to purchase healthy food, and the lack of food [9]. Previous evidence shows that HIV/AIDS populations in Africa experience higher levels of food insecurity, where malnutrition, specifically undernutrition, is already endemic and can pose more challenges for PWH in attaining optimal prevention and management as well as increasing severity of HIV/AIDS symptoms [10]. Undernutrition is present when an individual has suboptimal nutrition that limits health and growth [11].

Over 100 million Africans were food insecure in 2020, a number that is expected to increase by 60% in 2021 [12]. Employing a synergistic epidemic lens via the syndemics framework, food insecurity coexists and overlaps with the significant prevalence of HIV/AIDS in various regions in Africa. PWH experiencing food insecurity are likely to experience hunger, coupled with the side effects of ART, in the context of competing resources, which could increase the chances of treatment non-adherence [13]. In the context of food insecurity, evidence shows that HIV is a strong predictor of malnutrition, potentially increasing vulnerability to other comorbidities such as tuberculosis (TB) [14]. Undernutrition weakens immunity, thereby increasing the chances of developing active TB; most individuals with active TB are in a catabolic state, and as such suffer from weight loss/wasting [11]. Moreover, among PWH, food insecurity is a risk factor for non-adherence to HAART, further exacerbating negative health outcomes among PWH [15]. Socioeconomic factors such as poverty catalyze food insecurity [16], and there is evidence that shows that various nutritional deficiencies, as a by-product of malnutrition, could contribute to the pathogenesis of HIV through the drop in the count of immune cell surface glycoproteins, known as cluster differentiation 4 (CD4) [17].

Synergistically, there is also an abundance of evidence that captures the harmful interactions between HIV/AIDS and TB in Africa [18], where the overlap and clusters of both diseases interact synergistically to negatively impact the health of populations; these interactions are driven by various social, cultural, and economic factors [19]. The prevalence of all HIV/AIDS and TB co-infection was 31.25% in African countries [20]. As of 2017, 72% of global HIV-associated TB cases were in Africa [21]. In 2020, 214,000 TB deaths (14.1%) came from HIV-positive individuals [22]. Nevertheless, Sub-Saharan Africa accounts for 25% of new global TB cases [23]. The vulnerability to TB among PWH has positioned TB as the leading cause of mortality among PWH [23].

The double burden of HIV/AIDS and TB has also been linked to malnutrition, unemployment, substance use disorder, poverty, and homelessness [24]. When coupled with highly prevalent food insecurity in Africa, HIV/AIDS and TB co-infection can interact synergistically to worsen negative health outcomes among PWH. This interaction can be further established because active TB has a strong nutritional impact, presenting as weight loss/wasting in infected individuals with active TB [25]. Nutritional symptoms such as wasting and malnutrition are highly prevalent in symptomatic TB infections and can also be seen as symptomatic indicators of food insecurity [25,26]. This can be explained via a bidirectional relationship between nutritional symptoms and active TB infections, where the immunopathology of TB reduces the appetite of infected individuals, thereby limiting their ability to consume healthy quality and quantity of food [25].

In the African context, it is plausible that food insecurity is a nutritional risk factor for or a symptom of active TB infection due to the strong nutritional influence of TB infection among infected populations. Strong evidence suggests that low body mass index (BMI) (as a result of food insecurity) is associated with increased TB incidence; however, the biological causal mechanism is unknown [27]. Research also suggests that macronutrient supplementation among food insecure individuals with TB may improve health outcomes, although high-quality evidence is currently lacking [28]. Insufficient to lack of macronutrients (i.e., carbohydrates, fats, and proteins) are more telling signs of food insecurity, as the human body relies on these nutrition sources for required energy needs [29].

This review aimed to synthesize published evidence on the characteristics of the synergistic relationship between HIV/AIDS and TB co-infection, and food insecurity in Africa. Given the high prevalence of HIV, TB, and food insecurity in Africa, this review would broaden the holistic understanding of the influence of highly prevalent contextual factors (i.e., food insecurity) on clinical and other health-related outcomes among the HIV/AIDS and TB co-infected population in Africa.

## 2. Materials and Methods

We conducted a scoping review, using the systematic approach, guided by a consolidated checklist of the Arksey–O’Malley framework and the Preferred Reporting Items for Systematic Reviews and Meta-Analyses (PRISMA) checklist (Supplementary Materials File S1), to ensure a comprehensive appraisal of included articles [30,31].

### 2.1. Inclusion and Exclusion Criteria

Studies were included if they met the following inclusion criteria: (1) conducted in Africa, (2) included both HIV and TB populations (either on prevalence, treatment, screening, referral to care, retention in care, or treatment adherence), (3) addressed food insecurity from the standpoint of macronutrient deficiency, using either narrative or quantitative indicators of assessing food insecurity, (4) empirical population studies of adults and children, and (5) published in English or had translated versions available for non-English articles. No limitation was placed on study design and publication year except systematic reviews and commentaries.

Additionally, studies were excluded if they: (1) addressed only HIV or TB populations, (2) discussed food availability/insecurity from the standpoint of micronutrient deficiency, (3) abstract-only or protocol papers, and (4) were not published in English or did not have English translated versions available.

### 2.2. Information Sources

We sourced information from the following databases: PubMed, Cumulative Index to Nursing and Allied Health Literature (CINAHL), Web of Science (all databases), Embase, Global Health, APA PsycINFO, and Cochrane Library for studies. The gray literature from the following databases was used: PubMed, Global Health, Medline, Embase, Cochrane Library, LILACs, Africa Wide, WHOLIS, abstracts from the International Aids Society (IAS) Online Resource Library from 2006 to 2007, and the HIV Implementers meetings from 2007 to 2020 as well as international conferences on non-communicable diseases (NCDs) and firsthand correspondence from implementers.

### 2.3. Search Strategy

A comprehensive search strategy was developed to identify studies that met the predefined inclusion criteria. We included filters to exclude non-human studies and reviews. Multiple primary and secondary study designs were included (randomized controlled trials (RCTs), cohort, cross-sectional, case–control, qualitative studies, quasi-experimental, pre–post, before/after studies). The article search was conducted on 28 April 2021. The full search strategy is provided in Appendix A.

### 2.4. Selection Process

All citations were downloaded to Covidence for screening [32]. Retrieved articles were subjected to review and assessment separately by two pairs of reviewers to reduce bias. Each pair of reviewers screened the same set of articles independently to determine if the inclusion criteria were met by assessing the title or title and abstract. Following title and abstract screening, full-text reviews were conducted by two pairs of reviewers to confirm if all eligibility criteria were met. Full-text analyses were also carried out to rule out article abstracts that were ambiguous. Disagreements were resolved by a third independent reviewer.

### 2.5. Data Collection Process

After confirming that the preselected articles met the criteria, a Google Form was utilized to extract the data. Outcomes were collected by utilizing data items and investigators were contacted for further information regarding their research strategies. Inconsistencies in data were subjected to the judgment of a third independent reviewer and final articles were ultimately chosen by consensus.

### 2.6. Data Items

Data were collected on the presence of food insecurity using either a validated tool such as the Household Food Insecurity Access Scale (HFIAS) [33], which has a Cronbach’s α of 0.83–0.90 and a score range of 0–27 and four categories of food insecurity: food secure, mildly insecure, moderately insecure, and severely insecure, or reported narrative about food insecurity—including contextual information where food insecurity is endemic, or nutritional metrics such as BMI or malnutrition [34]. The HFIAS was developed by the USAID Food and Nutrition Technical Assistance II Project (FANTA), and the higher the score, the more food insecure it becomes [33]. Other data collected include the prevalence or incidence of HIV and TB in the sample population, study sample size, the type of intervention/programming administered, duration of intervention/programming, type of healthcare setting (community-based, primary healthcare centers, hospitals, pharmacies), the location of intervention/programming (local, peri-urban, or urban), type of implementer, if implementers were newly hired because of the interventions, existing or hybrid of new and existing staff of health settings, if implementers received training, if participants were adults, children, or both, primary and secondary study outcomes, factors that were reported to influence health outcomes pertaining to HIV and TB, clinical outcomes of HIV (i.e., CD4 count and viral suppression) and TB, treatment adherence, an association between food insecurity and HIV viral suppression, an association between food insecurity and TB progression, self-reported poor health, poor nutrient absorption, increase in cardiopulmonary or morbid diseases, increase in stress, and decrease in sleep, mental stress from food insecurity and HIV/AIDS and TB co-infection, behavioral risk factors, economic factors, and social factors.

### 2.7. Study Risk of Bias Assessment

The risk of bias assessment was conducted on all included studies, using a modified Google Form. Various risks of bias assessment tools were utilized to assess studies, depending on the study design. Randomized control trials were evaluated using the Cochrane risk-of-bias tool (Version 2) [35]. The Newcastle–Ottawa Scale (NOS) was used to assess cohort and case–control studies [36]. The Joanna Briggs Institute (JBI) Critical Appraisal Checklist was used to review cross-sectional studies [37]. Qualitative studies were analyzed using the corresponding Critical Appraisal Skills Programme (CASP) checklist [38]. The Mixed Methods Appraisal Tool (MMAT) version 2018 was utilized to assess mixed methods studies [39]. Two reviewers assessed each study independently and disagreements in the independent responses of the reviewers were resolved by a third reviewer.

### 2.8. Effect Measures

Effect measures did not apply to all of the data items of this review. For clinical outcomes of HIV and TB, effect measures included odds/risk ratios of the association between HIV, TB, and food insecurity, with food insecurity being the primary exposure and the primary outcomes being related to an individual’s HIV and TB status. HIV viral suppression was defined as less than 50 copies/mL, CD4 count was measured as cells/mm^3^ or cells/microliter, and HIV + TB prevalence or incidence was measured as a percentage.

### 2.9. Synthesis Methods

Data were synthesized using (1) a descriptive table of the included studies, (2) a narrative table of study outcomes related to food insecurity, HIV- and TB-related health outcomes, treatment adherence, behavioral risk, economic drivers, and influences, (3) reporting of descriptive analyses such as means, frequency, and range of quantitative measures, and (4) qualitative content analysis [40] and narrative description of the syndemic coupling between HIV/AIDS and TB co-infection and food insecurity. Studies were reviewed for reporting syndemic concepts directly or indirectly regarding food insecurity among individuals with HIV/AIDS and TB co-infection.

### 2.10. Reporting Bias Assessment

Assessing reporting bias due to missing data was determined by estimating the missing proportion in different studies and setting a threshold of less than 5% for an acceptable proportion of missing outcome data if the information was available.

## 3. Results

**Figure 1:**
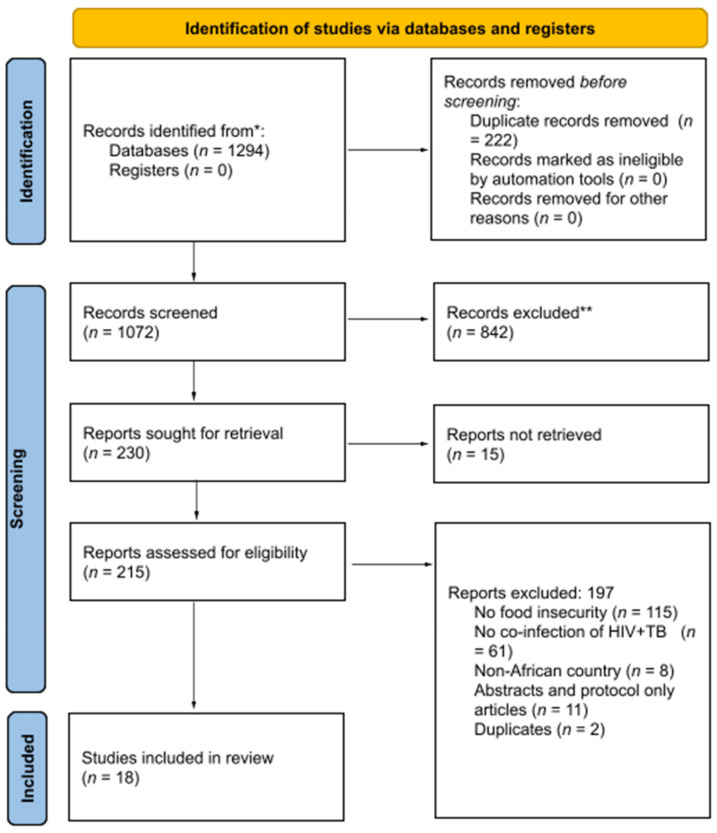
PRISMA study flowchart. This chart shows the systematic screening of studies, using inclusion and exclusion criteria to advance or eliminate studies for the review. Two stages of screening occurred: first, titles and abstracts were screened for 1072 studies, excluding 842 records; second, full texts were retrieved and screened for 215 out of 230 studies. Of these 215 studies, 18 studies made it to the final review. * Consider, if feasible to do so, reporting the number of records identified from each database or register searched (rather than the total number across all databases/registers). ** If automation tools were used, indicate how many records were excluded by a human and how many were excluded by automation tools.

An initial search retrieved 1294 articles and 1072 articles remained after removing duplicates. Screening of title and abstract further excluded 842 articles. Two hundred and thirty full-text articles were reviewed, of which 18 studies were included in the review. The screening, elimination process, and reasons for excluding articles are outlined in the PRISMA chart (Figure 1).

The studies comprised diverse sample sizes ranging from 9 participants to 11,784 participants. Eighteen studies were conducted across 11 African countries, with South Africa being the leading location, with four studies. Fourteen studies were conducted amongst adults, two studies were conducted among children only, and two studies looked at both children and adults. Studies were conducted in rural settings (*n* = 5 studies), in urban settings (*n* = 9 studies), in a peri-urban setting (*n* = 1 study), in sub-district community clinics (*n* = 1 study), primary health centers (*n* = 1 study), and hospitals (*n* = 1 study) (Table 1). The average study duration was 29.5 (SD: 57.0) months. Most of the studies were observational designs (*n* = 17 studies), with only one randomized implementation study.

### 3.1. Evidence of Food Insecurity in Individuals with HIV/AIDS and TB Co-Infection

Food secure households experience no issues with access and may rarely experience worry. Mildly food insecure households worry about having enough food often and may rarely have to eat not preferred/undesirable/monotonous food; moderately food insecure households have to give up quality food more often and may have to cut back on the quantity of food rarely or sometimes. Severely food insecure households experience cutting back on the quantity of food often and experience at least one of these severe conditions: going to bed hungry, running out of food, or going a whole day and night without eating [31].

Reporting of food insecurity was inconsistent across the included articles. Besides the well-validated HFIAS [14,41,46,56,57], authors evaluated food insecurity by diagnosing malnutrition using BMI values [14,47,49,50,52,53,54,58], mid-upper arm circumference (MUAC) [47,48,49], weight-for-height z-score [48], lean mass index (LMI) [51,52], fat mass index (FMI) [51,52], bioelectrical impedance analyzer (BIA) measurements [51,52], food energy/nutritional intake of macronutrients [43,45,51,54], dietary diversity score [53], weight gain or loss [14,41,51,53,57], clinical symptoms such as kwashiorkor, pellagra, and marasmus [43,45], qualitative reports of food availability [42,55], food sufficiency [42], or presence/absence of nutritional support [43,44,50]. A couple of the studies only reported a narrative description of food insecurity, without stating the measure used [42,44,55]. For instance, in studies that reported food insecurity-induced malnutrition, the adult average BMI estimates ranged from 16.5 kg/m^2^ to 18.5 kg/m^2^. BMI less than 18.5 kg/m^2^ was deemed as malnutrition, with BMI values less than 16 kg/m^2^ deemed as severe malnutrition amongst adults [50,52,54]. Amongst studies where children were observed (n = 4), weight-for-height z-scores were used to assess malnutrition [48]. In studies that reported food insecurity-induced malnutrition using BMI (n = 8), the adult average BMI estimate ranged from 16.2 kg/m^2^ to 23.3 kg/m^2^ [14,47,49,50,51,52,53,54]. Where weight was used to indicate food insecurity, weighing less than 18.2 kg (for girls) and 18.3 kg (for boys) in children indicated malnutrition [53]. Where MUAC was used to diagnose malnutrition, 96.4% of men (MUAC ≤ 24 cm) and 94.7% of women (MUAC ≤ 23 cm) were malnourished and, in malnourished children, mean MUAC was ≤11.5 cm [48,49]. In another study that used LMI and FMI to diagnose food insecurity-induced malnutrition, the cutoffs for low LMI and FMI corresponding to a BMI < 18.5 kg/m were as follows: LMI < 16.7 (kg/m^2^) for men and <14.6 (kg/m^2^) for women with corresponding FMI < 1.8 (kg/m^2^) for men and <3.9 (kg/m^2^) for women [52] (Table 2).

Among studies that used the Household Food Insecurity and Access Scale (HFIAS) to assess food insecurity (*n* = 5), all reported the presence of food insecurity [14,41,46,47,57] but only one study reported the actual HFIAS score, where the average score was 6 (range: 1–14) [41]. Another study at baseline started with 92% food insecurity at enrollment, which declined to 73% food insecurity, after the nutritional support and TB treatment initiative was administered for 6 months [14]. One study reported 51% of their participants being severely food insecure [47], another reported 48.5% of participants with HIV/AIDS and TB co-infection being food insecure, compared to 44.4% of participants without HIV being food insecure [57], and another reported 35.2% of their participants being food insecure, where food insecure households were significantly more undernourished (*p* = 0.0001) [46]. In one study, low BMI and LMI increased the hazard of death among HIV/AIDS and TB co-infected individuals, in addition to other risk factors including being older than 30 years old, being male, and having a history of weight loss [53]. In another study, adult participants were reported to gain from 0.6 kg to 1.9 kg at the end of the studies; children were reported to gain from 0.4 kg to 1.4 kg at the end of the studies, with 97% of adults and 57% of child participants having food insecurity-induced malnutrition [54]. Impaired nutritional energy was described as an imbalance in the intake of macronutrients, such as the overconsumption of starchy carbohydrates, and low intake of proteins, fruits, and vegetables [43,45,52,54,55]. Among studies that gave a narrative description of food insecurity, food insecurity was described as lack of food, not having enough food to eat, and not being able to afford food [42,44,55].

### 3.2. HIV- and TB-Related Outcomes in Co-Infected Individuals

Of the 18 included studies, 14 studies reported on HIV (*n* = 3) [41,51,52,53], TB (*n* = 2) [49,55], or both HIV- and TB-related (*n* = 9) [42,43,44,45,46,47,48,52,53] outcomes, in addition to food insecurity. Health and health-related outcomes reported among individuals co-infected with HIV and TB include morbidity [43,44,48,52], mortality [41,42,45,51,52,53,55], treatment outcomes (e.g., treatment non-adherence, access to treatment, initiated ART, treatment success, treatment failure, etc.) [42,44,51], weight loss/wasting [46,52,53], opportunistic infection [46,48], TB lung disease [47], and lethargy [49]. Having access to food helped people be cured of TB [42]. Significantly higher mortality and treatment non-adherence were observed among co-infected individuals compared to individuals with only one infection [42]. Moreover, economic, social, and health facility service barriers discouraged co-infected individuals from accessing and adhering to ART and TB treatments [44]. In addition, not all co-infected individuals were aware of their HIV or TB diagnosis and not all had received prior ART or TB treatment [51,57].

### 3.3. Treatment Adherence and Health Behaviors in Individuals Co-Infected with HIV and TB

Some studies (*n* = 7) documented HIV- and TB- related treatment adherence and health behaviors amongst co-infected individuals [14,41,42,44,51,56,57]. Treatment non-adherence was reported as the inconsistent and incorrect use of medication or treatment as prescribed by health providers, and was reported in several studies [14,42,44,51,56,57]; in one study, 31% of the individuals with HIV/AIDS and TB co-infection were reported as non-adherent to treatment [57]. Other documented related health behaviors included: if the patient was on treatment (e.g., medications such as ART) and had access to treatment [41,42,44,51]. The presence of food and nutritional support, whether in the form of nutrient-rich food, ready-to-use therapeutic food (RUTF), or a supply of food ingredients, supported high treatment adherence [14]. In some instances, individuals with HIV/AIDS and TB co-infection experienced more treatment defaults; however, having food or access to food supported TB cure [42]. Treatment adherence was impacted by economic conditions (i.e., lack of funds), social situation (i.e., family disapproval of visiting ART clinics), and health facility barriers (i.e., transportation) to accessing free ART and TB treatment [44]. Co-infection status and management of both infections exacerbated food insecurity [44].

We also found that treatment adherence was impacted negatively by social factors such as stigma and individual-level factors such as patients not prioritizing treatment, temporary relief felt by patients, and significant side effects of ART and TB treatment regimens [55]. Moreover, health facility factors that were significant barriers to treatment adherence among patients included delayed services and unhygienic conditions of facilities [55]. As a reported consequential side effect of treatment was hunger, food insecurity becomes an indirect barrier to treatment adherence, when the patient has no food to accompany the routine consumption of pills [55]. In a study where approximately 47% of the participants experienced mild to severe food insecurity, a little over a third of participants, who were mostly newly diagnosed with HIV, had never taken ART [57].

### 3.4. Economic Drivers of Food Insecurity in HIV/AIDS and TB Co-Infected Individuals in Africa

Some studies (*n* = 5) reported economic influences on food insecurity in HIV/AIDS and TB co-infected populations in Africa [43,44,46,51,54]. National-level economic collapse and crisis, coupled with high HIV prevalence, resulted in higher food insecurity and a high incidence of TB [43]. Co-infected participants experienced worsening food insecurity due to their illnesses, as they lost livelihood and experienced decreased productivity, coupled with mounting debt from increased spending on managing their conditions and purchasing appropriate nutritional support [44]. Unemployment amongst co-infected individuals was associated with a higher risk of malnutrition and food insecurity as lack of income limits individuals’ access to healthy food that supports better health outcomes [46,54]. A study conducted in South Africa found that most adults (96.5%) and children (57%) who lived in resource-poor settings experienced higher unemployment and HIV prevalence rates than the national averages, showing that unemployment might be a potential risk factor [54]. An additional economic driver of food insecurity reported was poverty [44,46,51]. Addressing these drivers through the provision of monthly food baskets for destitute patients, transportation assistance, and house rent coupled with psychosocial support in the form of a patient supporter resulted in a high percentage (90.2%) of co-infected individuals being on treatment [51].

### 3.5. Syndemic Coupling Linking Food Insecurity, Other Contextual Factors, and HIV/AIDS and TB Co-Infection in African Settings

Across the studies, there was no direct acknowledgment of syndemics as a framework for understanding interactions and effects between HIV/AIDS and TB co-infection and food insecurity. Nonetheless, food insecurity plays a significant role in HIV/AIDS and TB co-infection and is also associated with worsening health outcomes such as higher treatment non-adherence rates and mortality amongst affected individuals [42,44,45].

Food insecurity was reported among PWH as a precursor of TB infection, reported to induce atypical symptoms of pulmonary TB among co-infected individuals, or reported as a symptom of TB in an HIV population [16,43,50]. As a precursor, food insecurity preceded the rise in TB incidence when a country was in crisis or during dry seasons (a time of the year in Africa when rainfall is scarce or absent), which affect food supply [43]. By interacting with HIV, food insecurity-induced malnutrition caused atypical symptoms of pulmonary TB, especially when malnutrition was severe [50]. In other instances, food insecurity could be a suggestive symptom of TB in a co-infected adult population [47] or was reported to mask the presence of pulmonary TB in severely malnourished children, leading to underestimation of TB diagnosis [49]. A couple of studies also documented a high prevalence between food insecurity and HIV/AIDS and TB co-infected individuals [46,57] and the association between food insecurity and depression and anxiety in individuals with HIV/AIDS and TB co-infection [57]. The synergistic effect of food insecurity and HIV/AIDS and TB co-infection has been documented through indirect factors that exacerbated food insecurity as a barrier to positive health outcomes in co-infected individuals [43,44,46,54] and through the benefits of implementing initiatives that addressed food insecurity in co-infected populations [51]. Food insecurity was reported because of economic factors such as financial hardships, poverty, and unemployment [43,44,46,54], and the absence of nutritional support [56], which resulted in poor health outcomes. By implementing nutritional support programs such as monthly food baskets, providing nutrient-rich food, stipends, and providing psychosocial support such as patient supporters, the harmful effect of food insecurity in individuals with HIV/AIDS and TB co-infection is minimized and individuals were able to attain treatment success [14,51].

### 3.6. Risk of Bias Assessment

**Figure 2:**
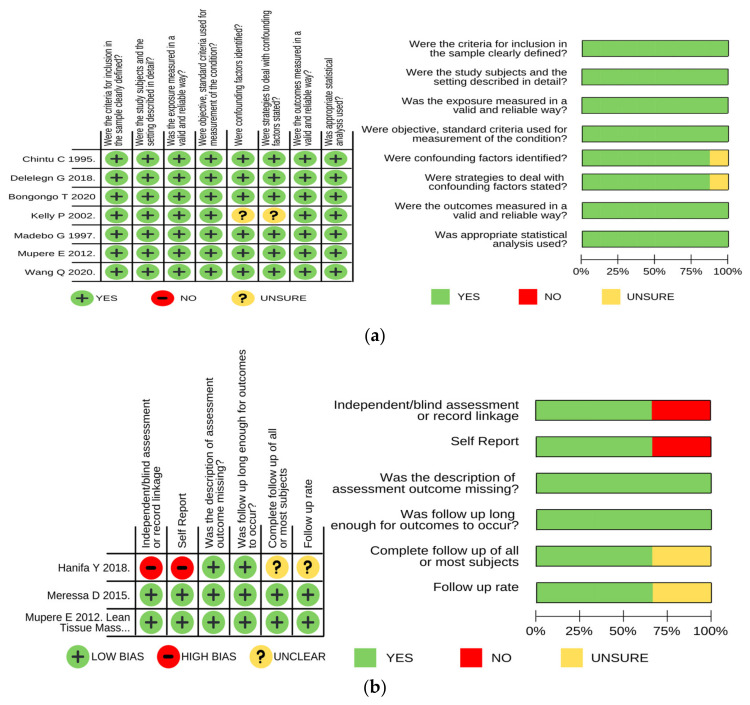

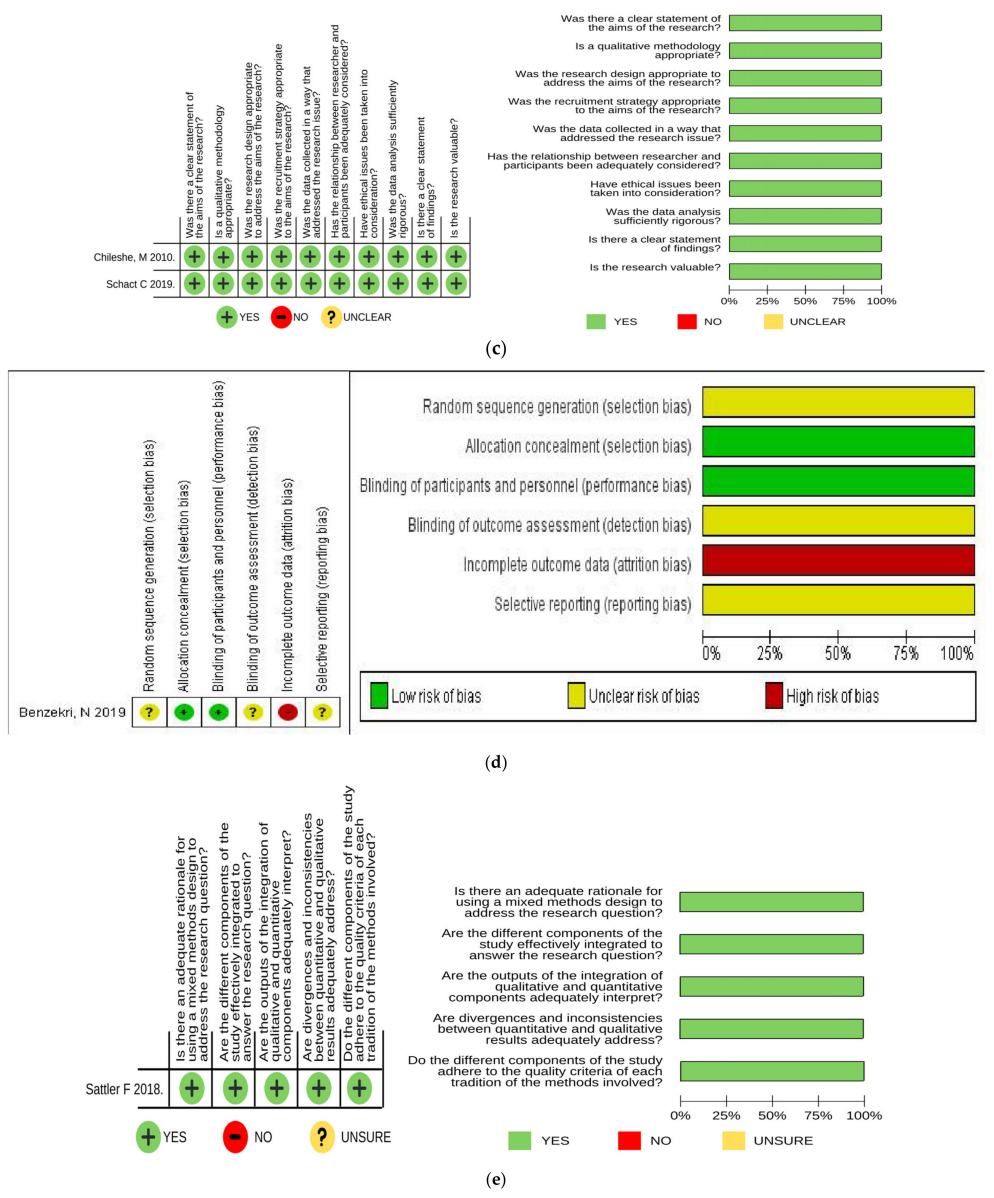
(**a**) Risk of bias assessment of cross-sectional studies (*n* = 7). At least 75% of all cross-sectional studies had a low risk of bias across all indicators for the quality of evidence gathered in the studies. (**b**) Risk of bias assessment of cohort studies (*n* = 3). Two of the three cohort studies exhibited a low risk of bias across all indicators for assessing the quality of evidence for cohort studies. (**c**) Risk of bias assessment of qualitative studies (*n* = 2). Across all indicators for assessing the quality of evidence, 100% of the qualitative studies had a low risk of bias in the evidence reported. (**d**) Risk of bias assessment of a randomized implementation study (*n* = 1). The singular randomized study only had a low risk of bias for 3 indicators (allocation concealment, blinding of participants, and blinding of personnel) out of 7 total indicators for assessing the quality of evidence. (**e**) Risk of bias assessment of a mixed methods study (*n* = 1). The singular mixed methods study had a low risk of bias across all indicators for assessing the quality of evidence for this type of study design.

The majority of the studies (*n* = 12) had a low risk of bias, indicative of the high quality of evidence (Figure 2a–e). Risk of bias of pre–post studies (*n* = 2) [41,54], a prospective observational study (*n* = 1) [49], and an ecological analysis (*n* = 1) [43] could not be assessed as there was no risk of bias assessment tools available for these study designs.

## 4. Discussion

We identified 18 studies that documented the syndemic relationship between food insecurity and HIV/AIDS and TB co-infection in an African population. Studies examined clinical and health-related outcomes and narrated the lived experiences of adults living with an HIV/AIDS and TB co-infection in both rural (27.7%) and urban settings (50.0%) in eleven African countries. Within the studies we examined, food insecurity was classified as malnutrition that was reported as a loss of weight, low BMI, and deficits in energy and protein intake [52]. In some instances, diseases of malnutrition such as wasting, kwashiorkor, marasmus, and pellagra were reported in affected individuals [45,46,52,55].

This review provides evidence of a syndemic relationship between food insecurity, economic influences, health-related behaviors, and HIV/AIDS and TB co-infection. The inconsistent assessment and classification of food insecurity within the context of the various studies were problematic, however, it does indicate that food insecurity is a driver of stress, and the lack of nutrition and calories in the absence of food provides opportunities for co-infections (e.g., TB) [44]. In addition, the stress around not being able to acquire food for oneself or the family unit can lead to engagement in high-risk behaviors.

### 4.1. Public Health Research and Policy Implications of Syndemic Coupling of HIV/AIDS and TB Co-Infection and Food Insecurity

Findings from this scoping review highlight the interconnectedness of multiple disease burdens and societal factors on affected individuals, especially against the backdrop of limited resources, socioeconomic disparities, or weak health systems. For instance, economic hardship, such as unemployment, was identified as a cause of food insecurity and subsequently a barrier to achieving treatment success among individuals with HIV/AIDS and TB co-infection [43,44,46,54]. On the other hand, individuals living with both HIV and TB reported that the burden of living with and managing an HIV/AIDS and TB co-infection resulted in the loss of income, unemployment, and decreased productivity, which in turn exacerbates food insecurity in their context [44]. Similar interconnected relationships between disease burden and societal factors have been documented in research on substance use disorders and chronic management of NCDs, such as mental health disorders [58,59]. For instance, a global analysis of mental health disorders shows data that associated the alarming increase in expenditure on mental health disorders, projected to reach 6 trillion dollars by 2030, as predominantly linked to loss of income due to chronic disabling traits of some mental health issues and treatment [58]. Specifically, about 49% of 120 countries rank household income as either the primary source or second most important source of funds to meet mental health expenditures [59].

Direct interventions that tackle one or more of the causes of a component of a syndemic interaction can have beneficial effects on the overall health outcomes and experience of individuals living with multiple burdens of diseases. In the case of this review, initiatives that minimize food insecurity such as the provision of nutritional support (such as RUTF and monthly food baskets) and providing economic assistance such as transportation fare and house rents improved the treatment outcomes of individuals with HIV/AIDS and TB co-infection [14]. Actionable solutions guided by a syndemic approach to tackling disease burden have been documented in other instances of multifactorial influences in populations experiencing multiple burdens of diseases. African countries such as Rwanda and South Africa have developed and implemented integrated delivery of health services for linked disease burden (HIV, TB, diabetes, mental health disorders), heavily influenced by social and structural factors such as economic hardships, stigma, and scarcity of trained health professionals to meet health needs [60]. These integrative programs ensure that access to needed healthcare services is easy for affected populations because they can access all their health needs at one location and/or be supported by community health worker for follow-up support, which promotes retention in care and treatment adherence [60].

Though findings from this review highlight salient effects of experiencing a cluster of comorbid conditions—in this case, food insecurity, HIV, and TB on health and wellbeing of individuals managing chronic conditions in Africa—the literature is scarce, and methodology is inconsistent. Only 18 articles captured syndemic interactions between food insecurity and prevalent infectious-to-chronic diseases of HIV and TB in African countries. The methodology for measuring food insecurity was inconsistent, as not all researchers utilized well-validated tools [33,61]. Future research efforts could be invested in the creation of a standardized list of indicators, which has been peer-reviewed and has a consensus amongst experts to improve the consistency of measures and the methodology for assessing food insecurity. For instance, multilateral organizational platforms such as the United Nations General Assembly or the Food and Agriculture Organization-specific meetings provide a scientific space for experts to tackle the issue of inconsistent measures for food security as an agenda item.

Across the studies, there was no direct acknowledgment of syndemics as a framework for understanding interactions and effects between HIV/AIDS and TB co-infection and food insecurity. This shows a missed opportunity in public health research to employ the syndemics framework in understanding complex interactions between the physiological presentations of diseases and the multifactorial socio-economic and environmental conditions that control or cause marked health and behavioral outcomes in subpopulations. Practical steps should be taken to include whole sections and panels on the syndemics framework in scientific conferences. This will allow researchers to present their work on the application of syndemics to understand the cluster of comorbid conditions of food insecurity, HIV/AIDS, and TB. Moreover, researchers can connect with others whose work interconnects with or could benefit from the use of a syndemics framework. In the policy sphere, health policy experts and researchers should accelerate the creation, adoption, and implementation of guidelines and technical packages that utilize a multisectoral and systems approach to outline, prioritize, and delineate action items in addressing the different dimensions (social, economic, and health) of the syndemic burden of food insecurity, HIV/AIDS, and TB, through an integrated policy and implementation vehicle. This recommendation can be modeled after policy tools such as the World Health Organization (WHO) NCD Multisectoral Action Plan (MAP) tool [62].

Besides treatment adherence, a majority of the included studies in this review did not document behavioral impact and/or influences on the syndemic interactions between food insecurity and HIV/AIDS and TB co-infection in affected individuals. Health and related behavior remain a critical component of HIV and TB health research. In this area of research, many interventions and policies are designed to target either the elimination of risky health behaviors or the facilitation of protective health behaviors [60,63]. This review further exposes missed opportunities within public health research and policy to employ a syndemic framework in identifying interventional pathways to encourage and facilitate healthy behaviors that increase the likelihood of treatment successes for populations with HIV/AIDS and TB co-infection in Africa.

### 4.2. Strengths and Limitations

This study used the rigorous PRISMA framework to review literature documenting syndemic interactions between food insecurity and HIV/AIDS and TB co-infection in Africa. Additionally, no study designs were restricted to ensure a full sweep of published data. We were able to capture relevant information from the observational studies that were included in the review. Included studies were restricted to English-only articles or available translations of non-English articles—it is possible the review omitted studies not published in English.

## 5. Conclusions

In African populations living with HIV/AIDS and TB co-infection, where food insecurity is endemic, syndemic relationships between these three conditions present as worsening clinical and health outcomes such as mortality, treatment default, depression, anxiety, and food insecurity-induced malnutrition amongst co-infected individuals [45,46,52,55,57], of which malnutrition was the most common outcome of experiencing food insecurity while living with an HIV/AIDS and TB co-infection. In other instances, food insecurity was reflected as a precursor to co-infection, especially with the onset/increased incidence of TB in an HIV prevalent population or observed to induce or be a symptom of pulmonary TB, especially in severely malnourished adults [43,50,58]. In children with HIV and TB, food insecurity, especially severe malnutrition, was seen to mask the diagnosis of pulmonary TB [49]. Economic, social, and facility-level factors influenced the pronounced negative impact of food insecurity on the health and lived experiences of individuals with HIV/AIDS and TB co-infection [43,44,46,54]. Direct tackling of food insecurity through nutritional support, economic relief, and psychosocial support minimized the harmful synergistic effects of being food insecure and living with HIV and TB [14,51]. It also influenced health-related behaviors, prominently treatment adherence, which was reflected in high treatment success in co-infected individuals [7]. Findings from this scoping review illustrate the interrelatedness of multiple disease burdens and social factors in affected individuals, especially in the presence of limited resources, socioeconomic disparities, or weak health systems. Direct interventions that address multiple factors that give rise to syndemic interactions can be advantageous for the overall health outcomes and experience of individuals living with multiple burdens of diseases; similar comprehensive strategies should be continued to be implemented for sustainable and effective outcomes. Across the studies, there was no direct acknowledgment of syndemics as a framework for understanding interactions and effects between HIV/AIDS and TB co-infection and food insecurity. The lack of a syndemic focus in the literature may be contributing to poor conceptualization, implementation, and lack of relevant measures. Food insecurity is a serious human rights and public health issue, and one that merits special consideration when working to address the double burden of HIV/AIDS and TB in Africa.

## Figures and Tables

**Table 1:** Description of included studies.

Author (Year)	Location/Setting and Population	Study Objective	Study Duration (months)	Total Sample Size	Number of Patients with HIV and TB Co-Infection	Study Design	Delivery/Administration Method	Primary Outcomes
Bakari et al. (2013) [41]	Location: Urban primary health center in Tanzania Population: Adult women	“Determine the prevalence of low BMI in co-infected TB-HIV women Quantify specific deficits in energy and protein intake Assess food insecurity Assess changes in nutritional status during treatment”.	12	43	43	Pre–post anti-TB treatment study	Research staff	“HIV-positive women with TB have substantial 24-h deficits in energy and protein intake, report significant food insecurity and gain minimal weight on anti-tuberculosis treatment”.
Benzekri et al. (2019) [14]	Location: Urban hospitals in Senegal Population: Adults	“Compare the feasibility, acceptability, and potential impact of implementing two different forms of nutrition support for HIV-TB co-infected adults”.	13	26	26	Randomized controlled trial	Nurses, physicians, non-healthcare individuals	“Temporary nutrition support during the critical months of treatment against active TB, could contribute to improved adherence and treatment completion, and subsequently, improved clinical and socioeconomic outcomes”.
Bongongo et al. (2020) [42]	Location: Sub-district community-level hospitals in South Africa Population: Adults	“Determine the influence of patients’ living conditions on TB treatment outcomes”.	Not stated	180	83	Cross-sectional study	Research staff	“Tuberculosis treatment outcomes showed very little difference between where food security was and where there was little or no food security. This survey shows a high death rate (26.5%) and also a high default rate (31.3%) amongst TB respondents that are HIV-positive”.
Burke et al. (2014) [43]	Location: Hospitals in rural settings in Zimbabwe Population: Adults and children	Authors hypothesized that in the setting of high HIV prevalence, “widespread food insecurity would lead to a rise in TB incidence in Zimbabwe, as such performed an ecological analysis of the TB incidence during crisis year (2008–2009)”.	144	11,784	8838	Ecological study	Research staff	Incidence of TB increased during crises or the dry season when food insecurity was highest.
Chileshe et al. (2010) [44]	Location: Community-level hospitals in rural settings in Zambia Population: Adults	“Highlight barriers that poor rural Zambians co-infected with tuberculosis (TB) and HIV and their households face in accessing ART Account for patient outcomes by the end of TB treatment and beyond”.	10	9	7	Ethnographic case study	Research staff, nurses, counselors	Economic barriers included: “being pushed into deeper poverty by managing TB, rural location, absence of any external assistance, and mustering time and extended funds for transport and ‘special food’ during and beyond the end of TB. In the case of death, funeral costs were astronomical” [43]. Social barriers included: “translocation, broken marriages, a subordinate household position, gender relations, denial, TB/HIV stigma and the difficulty of disclosure”. Health facility barriers involved “understaffing, many steps, lengthy procedures, and inefficiencies (lost blood samples, electricity cuts)”.
Chintu et al. (1995) [45]	Location: Hospitals in urban settings in Zambia Population: Hospitalized children	“Assess the impact of HIV-1 on common childhood diseases Determine the pattern of HIV-1 seroprevalence and mortality”.	9	42	42	Cross-sectional Study	Research staff	Prevalence of common childhood diseases and death caused by HIV status among children with any of the recorded childhood diseases.
Gebremichael et al. (2018) [46]	Location: Community-level, primary health centers, and hospitals in rural settings in Ethiopia Population: Adults	“Determine food insecurity and nutritional status and contextual determinants of malnutrition among people living with HIV/AIDS”.	2	512	63	Cross-sectional study	Community health workers	The findings revealed a high prevalence of malnutrition and household food insecurity among people living with HIV/AIDS on antiretroviral therapy.
Hanifa et al. (2018) [47]	Location: Primary health centers in the Gauteng Province of South Africa Population: Adults	Identify the causes of symptoms suggestive of TB among people living with HIV.	3	103	14	Cohort study	Research staff, physicians	Post-TB chronic lung disease and food insecurity were the main diagnoses for symptoms suggestive of TB in our population of HIV clinic attendees, and diagnoses were assigned for more than 90% of participants.
Kelly et al. (2002) [48]	Location: Rural communities in Zambia Population: Adults	To examine the relationship between morbidity, nutritional impairment, and CD4 count in patients with HIV infection.	2	186	11	Cross-sectional study	Research staff	The findings suggest that “effective treatment of opportunistic infection is likely to be important in preventing or reversing nutritional failure, even when food availability is limited”.
LaCourse et al. (2014) [49]	Location: Hospitals in peri-urban settings of Malawi Population: Children	Determine pulmonary tuberculosis prevalence among the hospitalized severely malnourished.	2	300	52	A prospective observational study	Hospital staff and patient guardians	Only 2 of 300 screened patients had a positive cultured confirmed positive pulmonary TB diagnosis.
Madebo et al. (1997) [50]	Location: Hospital in an urban setting in Ethiopia Population: Adults	Examine the influence of HIV infection and malnutrition on the radiological and clinical features of pulmonary TB.	6	239	48	Cross-sectional study	Research staff	“HIV-positive TB patients had significantly more oral candidiasis, diarrhea, generalized lymphadenopathy, skin disorders, neuropsychiatric illness, hilar lymphadenopathy, but less cavitation and upper lung lobe involvement. The size of the Mantoux was associated with HIV infection and malnutrition. Malnutrition and HIV infection both contribute to the atypical presentation of pulmonary tuberculosis. The risk of such atypical presentation is particularly high among the severely malnourished HIV-infected patients”.
Meressa et al. (2015) [51]	Location: Community-level hospitals in an urban setting in Ethiopia Population: Adults	“Determine the patient-related (or clinical) and programmatic factors associated with successful multidrug-resistant TB treatment outcomes in a highly resource-constrained setting”. “All patients received a monthly food basket. After assessment of the patient’s living conditions, those found to be vulnerable due to extreme poverty were provided economic assistance for transport, additional food, and house rent if needed throughout therapy”.	60	612	133	Cohort study	Existing nurses, clinical staff of the health facilities, and family treatment supporters	“Though nearly half of the cohort had severe malnutrition (BMI < 16), overall, 64.7% were cured, and 13.9% completed treatment for a combined treatment success of 78.6%”. “Amongst co-infected participants, 69.9% had treatment success. In total, 5.9% of patients were lost to follow-up, 1.6% failed treatment and 13.9% died. The presence of nutritional, social, and economic support during study duration contributed to treatment success”.
Mupere et al. (2012) (Low Nutrient) [52]	Location: Hospitals in an urban setting in Uganda Population: Adults	This cross-sectional study “was conducted to establish the relationship between nutrient intake and body wasting and between nutrient intake and severity of tuberculosis disease at the time of tuberculosis diagnosis”.	7	131	31	Cross-sectional study	Research staff, health facility staff, nurses, nutritionists	The study found that “the average 24-h nutrient intake varied by the severity of tuberculosis disease, but not by tuberculosis disease or HIV status nor by nutritional status”. The findings also suggest that “in the face of tuberculosis disease, nutrient intake is reduced among patients with more severe disease regardless of HIV infection. In the absence of tuberculosis, nutrient intake was affected by gender, and not HIV infection”.
Mupere et al. (2012) (Lean Tissue) [53]	Location: Community clinics in the Kampala District of Uganda Population: Adults	“The study was conducted to assess the impact of wasting on survival in tuberculosis and HIV patients using precise height-normalized lean tissue mass index (LMI) estimated by bioelectrical impedance analysis and BMI”.	204	747	At least 99	Retrospective cohort study	Research staff	“Both low BMI and low LMI at tuberculosis diagnosis were associated with poor survival in univariate and multivariable Cox proportional hazards regression analyses”. “The unadjusted hazard ratio (HR) for death in a univariate model among patients with low BMI at diagnosis compared to patients who had normal BMI was 1.80 (95% CI: 1.23, 2.64). Similarly, the unadjusted HR for death among patients with low LMI compared to patients who had normal LMI was 2.34 (95% CI, 1.14, 4.80). Other univariate factors that were significantly associated with increased relative hazards of death included male gender, older age group >30 years, HIV seropositive status, and history of weight loss”. “The HR for death among patients with low BMI at presentation was 1.83 (95% CI, 1.24, 2.71) after adjusting for age, HIV, prior smoking status, the extent of disease on chest x-ray and history of weight loss”. “Men with low BMI at presentation had a greater risk of death compared with men who had normal BMI (HR = 1.70; 95% CI: 1.03, 2.81). Among women, those presenting with a low BMI compared with a normal BMI had a comparable risk of death (HR = 1.83; 95% CI: 0.96, 3.50)”. “Other factors that were associated with the risk of death in this model included older male gender, HIV seropositive status, and history of weight loss”.
Rudolph et al. (2013) [54]	Location: Community-level primary health centers in the urban setting of South Africa Population: Adults and children	“The aims of this 3-month pilot study with crèche children and adult TB patients in Alexandra, South Africa, were to generate baseline data on nutritional status, assess the impact of a fortified supplementary food (e’Pap) on nutritional status, and to evaluate the sensitivity and validity of non-invasive indicators of nutritional status”.	3	153	58	Pre–post pilot study	Research and health facility staff, community health workers	“Adult females were younger (mean age 34 years, STD 10 years) than males (mean age 40 years, STD 9 years). The mean age for female children was 4.6 years (STD 0.8 years) and for male children 4.7 years (STD 0.9 years). The median household size was 4 (Q1–Q3 3–4)”. “Unemployment was high among recruited adult participants-69% among males and 59% among females. 8 participants withdrew from the study reporting side effects like rashes, mouth sores, and vomiting. It was not possible to link the side effects to any particular cause, including consumption of the supplement”.
Sattler et al. (2018) [55]	Location: Hospitals in Kenya and South Africa Population: Adults	This study “hypothesized that greater malnutrition and/or inflammation when initiating treatment is associated with an increased risk for death”.	12	51	51	Retrospective case–cohort study	Research staff	“For the cohort of 51 participants, the 33 who survived were not different from the 18 who died. 14% of participants had BMI < 16.5 kg/m^2^ and 33% had BMI 16.5–18.5 kg/m^2^. The causes of death and week of death (in parentheses) in the ensuing year after randomization were disseminated: tuberculosis (2, 4), gastroenteritis (3, 5, 11), pulmonary tuberculosis (4, 10), acute renal failure (5), bacterial pneumonia (8, 34), cryptococcal meningitis (9), bacterial meningitis (13), bacterial sepsis (14), peritonitis (11), intracranial hypertension (16), gunshot wounds (24), tuberculous meningitis (40), and no information (23). Death could not be related to treatment assignment (early versus deferred ART). For measures of nutrition, hazard ratios for average BMI, low BMI, serum hemoglobin, serum albumin, and leptin were not significant risk factors for death, although there was a trend for increased risk of death with advancing age”. “Increased risk of death was significantly associated with CRP, but the only pro-inflammatory cytokine associated with a significant risk of death was IFNγ. MCP-3 was associated with risk of death, but MCP-1 was not”. “Global measures of the innate and adaptive immune responses were both strongly associated with risk of death”.
Schact et al. (2019) [56]	Location: Primary health centers in rural settings in Mozambique Population: Adults	“The purpose of this study was to elicit Mozambican patients with drug-sensitive TB (DS-TB), TB/HIV and Multidrug-resistant tuberculosis (MDR-TB) understanding and assessment of the quality of care for DS-TB, HIV/TB and MDR-TB services in Mozambique, along with challenges to effectively preventing, diagnosing, and treating TB”.	1	51	19	Qualitative study	Research staff	Themes from focus groups were classified under “(1) TB knowledge; (2) barriers to accessing services (including treatment); (3) barriers to treatment adherence, and (4) Suggestions for improvement for TB”. “Respondents identified numerous challenges including delays in diagnosis, stigma related with diagnosis and treatment, long waits at health facilities, the absence of nutritional support for patients with TB, the absence of a comprehensive psychosocial support program, and the lack of overall knowledge about TB or multidrug-resistant TB in the community”.
Wang et al. (2020) [57]	Location: Primary health centers in urban settings of Botswana Population: Adults	“To determine the association between food insecurity and HIV infection with depression and anxiety among new tuberculosis (TB) patients”.	12	180	99	Cross-sectional study	Research staff	“Among those who were HIV co-infected, the median duration since HIV diagnosis was 43 months, and 31(31.3%) had never taken ART, of whom 90% were newly diagnosed with HIV at or within one month of study enrolment”. “Higher prevalence of depression and anxiety in moderate to severe food insecure HIV-TB co-infected participants. Among HIV-co-infected participants, the estimates were similar in the bivariate analysis for depression (crude PR = 2.41; 95% CI 1.28, 4.55) and anxiety (crude PR = 1.54; 95% CI 1.04, 2.27). Accounting for ART status and CD4 count closest to the time of TB diagnosis, food insecurity continued to be associated with increased symptoms of depression (adjusted PR = 2.33; 95% CI 1.24, 4.38) and anxiety (adjusted PR = 1.53; 95% CI 1.03, 2.26) in the multivariable model”.

**Table 2:** Evidence and related outcomes of food insecurity among PWH with TB.

Author (Year)	Food Insecurity-Related Reports (HFIAS, Nutritional Metrics, or Narrative)	HIV-Related Health Outcomes	TB-Related Health Outcomes	Medication/Treatment Adherence or Behavior	Economic Drivers Influencing Food Insecurity	Was Intervention Successful (Y/N/NA)
Bakari et al. (2013) [41]	HFIAS average score 6 (range: 1–14)	“There was one reported death: a patient with a baseline BMI 21 kg/m^2^ and CD4 120 cells/μL who had gained 4 kg at 4 months, but remained sputum-positive, died at 5 months due to an undiagnosed febrile illness”.	NR	Twenty women (47%) were on ART but adherence was not reported.	NR	NA
Benzekri et al. (2019) [14]	Used HFIAS and indicators of nutritional status included weight, BMI, and percent malnourished. “The median weight increased from 50 kg at enrollment to 55 kg at month 6 and the median BMI increased from 17.3 kg/m^2^ to 19.3 kg/m^2^. At enrollment, 58% of subjects were malnourished versus 35% at month 6”.	NR	NR	“Overall, 7-day adherence, 4-week adherence, and medication possession exceeded 95% for both ART and TB treatment. Adherence did not differ between those who received ready-to-use therapeutic food (RUTF) and those who received food baskets”.	NR	Y
Bongongoet al. (2020) [42]	Had sufficient food daily for the past 12 months. “More participants (*n* = 124; 68.8%) were able to have at least one meal per day for the past 12 months. In all, 57 participants (45.9%) amongst those who had food were cured of TB”.	“More HIV negative participants (*n* = 33; 67.4%) were cured; more deaths (*n* = 22; 26.5%) and defaults (*n* = 26; 31.3%) were noted amongst HIV-positive patients”. “Association between TB patients on treatment who were cured, died or defaulted, when compared with their HIV status, was indicated by *p* = 0.001, 0.02 and 0.03 respectively”.	“57 participants (45.9%) amongst those who had food were cured. Whilst comparing the TB treatment outcomes and food security, an association of statistical significance in the group of relapses was noted, with *p* = 0.029”. “The effect of exposure to cigarette smoke in respondents’ families (passive or active) was evident. Of the 180 respondents with TB, 75 (67.6%) of the respondents who were not exposed to cigarette smoke were cured (*p* = 0.0001), whilst 45 (65.2%) of the respondents who were exposed to cigarette smoke died”.	“More deaths (*n* = 22; 26.5%) and defaults (*n* = 26; 31.3%) were noted amongst HIV-positive patients. Association between TB patients on treatment who were cured, died, or defaulted, when compared with their HIV status, as indicated by *p* = 0.001, 0.02 and 0.03 respectively”.	NR	NA
Burke et al. (2014) [43]	“Nutritional deficiencies reported include: The prevalence of kwashiorkor most significantly increased between 2001 (130 cases) and 2008 (239 cases) (*p* < 0.01). The prevalence of pellagra also rose between 2001 (31 cases) and 2004 (118 cases) (*p* < 0.01) and remained at this elevated level thereafter. Diarrhea, both with and without dehydration, increased over time (*p* < 0.01)”.	“Antenatal clinic HIV seroprevalence at HH decreased between 2001 (23%) to 2011 (11%) (*p* < 0.001). 75% of TB incidence in HIV population”.	“At the Howard Hospital (HH) in northern Zimbabwe, TB incidence increased 35% in 2008 from baseline rates in 2003–2007 (*p* < 0.01) and remained at that level in 2009. Murambinda Hospital (MH) in Eastern Zimbabwe also demonstrated a 29% rise in TB incidence from 2007 to 2008 (*p* < 0.01) and remained at that level in 2009. Data collected post-crisis at HH showed a decrease of 33% in TB incidence between 2009 to 2010 (*p* < 0.001) and 2010/2011 TB incidence remained below that of the crisis years of 2008/2009 (*p* < 0.01)”.	NR	Economic collapse and crisis, declining GDP per capita, a high prevalence of HIV.	NA
Chileshe et al. (2010) [44]	Narrated description: “Poverty was not regarded as a new phenomenon but embedded in seasonal patterns with food insecurity worse from October through to March. Illness and death were pinpointed as one of the main causes of current food shortages [43]”. Results: “The food insecurity of all six households had deepened by a period managing TB owing to loss of livelihood, assets, and income, a dip in productivity, mounting debt, and the cost of transport and requirements for ‘‘special food’’. None of the households received food aid or any other form of material assistance from the government, churches, or NGOs. All the seven co-infected participants spoke about their hunger and the need to take the medication with food. It was striking how the household diet had changed because of TB more fish, eggs, meat, soft drinks, and fruit were purchased; a sharp contrast to the normal household diet of vegetables and maize meal. For those co-infected and on ART, needing to eat to get well and take treatment was emerging as a more long-term problem”.	Out of the seven co-infected patients, two died before completing TB treatment.	“By the end of TB treatment, outcomes were mixed; two co-infected patients had died, three had started ART and two had yet to start ART”.	“Despite the free provision of both TB treatment and ART through government health services, co-infected patients faced economic, social, and health service facility barriers to accessing ART. In the study, two individuals reported being on ART but one stopped in 2008”. “Several of the participants reported not being able to access and adhere to treatment due to difficulty in being able to make it to the ART clinic due to family disapproval and transportation/economic barriers”.	“The food insecurity of all six households had deepened by a period managing TB owing to loss of livelihood, assets, and income, a dip in productivity, mounting debt, and the cost of transport and requirements for ‘special food’. Six of the seven co-infected participants had relocated whilst sick, five moving from town and having to leave their livelihoods behind. All TB patients were unable to contribute to the household living during their search for a diagnosis (which took between 220 months) and for at least four months into TB treatment; primary caregivers often found it hard to make a living just before and after TB diagnosis when patients were often extremely sick and required constant care or were admitted into the hospital. Five of the six households said that TB illness had disrupted their farming activities and made them less productive, with three households recording a drop in the maize they harvested and two households recording no harvest in 2006–2007”.	NA
Chintu et al. (1995) [45]	Use of nutritional metrics. Protein–energy malnutrition was reported as marasmus, kwashiorkor, and marasmic-kwashiorkor.	“94 children (40.5%) with malnutrition had HIV, 42 children (68.9%) with TB had HIV. 34/94 (36.2%) of children with malnutrition died from HIV; 7/42 (16.7%) of children with TB. The overall mortality among HIV-seropositive children (19 percent) was significantly higher than those who were HIV-seronegative (9 percent) (*p* < 0.001; overall odds ratio = 2.13; 95 percent CI = 1.49, 3.04)”.	61 children with TB died.	NR	NR	NA
Gebremichael et al. (2018) [46]	HFIAS score 27	HIV-induced immune impairment, increased risk of opportunistic infections, worsening of nutrition status.	Appetite loss, weight loss, wasting.	NR	“This study revealed that unemployed PWH were more likely to be undernourished compared with employed counterparts. The higher risk of developing malnutrition in unemployed subjects found in this study is supported by findings of other studies where unemployment promotes poverty, which in turn limits the ability of an individual to expend money for food consumption due to low income”.	NA
Hanifa et al. (2018) [47]	“HFIAS score: 50% (53 individuals) had severe food insecurity; On ART = 23 (46.0%); Not on ART = 30 (56.6%) Total average = 53 (51.5) HFIAS score: moderate food insecurity: On ART = 11 (22.0%); Not on ART = 8 (15.1%) Total average = 19 (18.5%) Weight loss due to severe food insecurity: *n* = 20 (19%)”.	“50/103 were pre-antiretroviral therapy (ART) and 53/103 were on ART; Seventy-two (70%) had 75% measured weight loss and 50 (49%) had cough. The most common final diagnoses were weight loss due to severe food insecurity (*n* = 20, 19%), TB (*n* = 14, 14%: confirmed *n* = 7; clinical *n* = 7), other respiratory tract infection (*n* = 14, 14%) and post-TB lung disease (*n* = 9, 9%)”.	Post-TB lung disease (*n* = 9, 9%).	NR	NR	NA
Kelly et al. (2002) [48]	“Height and BMI were low by comparison with norms in industrialized countries (Lentner, 1984), suggesting lifelong undernutrition in the population as a whole. Mean height was 1.58 m in women and 1.69 m in men (*p* < 0.0001). Mean BMI was 22.0 kg/m^2^ in women and 19.9 in men (*p* < 0.0001). MUAC also differed: mean MUAC was 27.3 cm in women and 26.3 in men (*p* = 0.04). BMI and MUAC were reduced in HIV seropositive adults with OIs”.	“65 participants were HIV positive; HIV seroprevalence was comparable to previous estimates of 22–35% for Lusaka. 33 (51%) of 65 HIV seropositive adults reported symptoms compared to 39 (32%) of 121 HIV seronegative adults (OR 2.2, 95%CI 1.1–4.2; *p* = 0.02) or 21 (28%) of 75 who were not tested (OR 2.7, CI 1.2–5.7; *p* = 0.01)”.	“The most frequently diagnosed clinical conditions in HIV-infected individuals were TB (*n* = 11). CD4 counts of less than 200 cells/L were found in 6/11 individuals with TB. Fourteen cases of TB were discovered including two who did not have HIV tests, five of which had previously been diagnosed”.	NR	NR	NA
LaCourse et al. (2014) [49]	“Enrollees were required to meet WHO severe acute malnutrition criteria for children aged 6–60 months with weight-for-height z-score ≤−3 standard deviations below the median, mid-upper arm circumference (MUAC) ≤115 mm, or bilateral pedal edema”.	NR	Lethargy/fatigue	NR	NR	NA
Madebo et al. (1997) [50]	“187 (77.9%) of the patients had a BMI < 18.5. Based on BMI assessment, 44 (18.2%)) were mildly, 39 (16.1%) moderately and 104 (43%) were severely malnourished. Based on MUAC assessment 161/167 (96.4) of men (MUAC ≤ 24 cm) and 71/75 (94.7%) of women (MUAC ≤ 23 cm) were malnourished. The mean BMI was 16.2 (SD 2.6) and 16.6 (SD 2.4) for HIV-positive and -negative patients, respectively”.	NR	NR	NR	NR	NA
Meressa et al. (2015) [51]	“Provision of a monthly food basket and in some extreme cases of poverty, provision of economic assistance for transport, additional food, and house rent if needed throughout Therapy. The median body mass index (BMI) was 16.6 (IQR 14.8–19.1). Treatment failure or death among MDR-TB patients was higher in patients who had severe malnutrition (BMI < 16 kg/m^2^) (15.1% vs. 6.8%, *p* = 0.003)”.	“Treatment success rates were higher for HIV-uninfected compared with HIV-infected individuals (81.0% vs. 69.9%, *p* = 0.008). Treatment failure or death was higher in HIV-infected patients”.	NR	“Of the 133 HIV-co-infected patients, 120 had begun ART before enrolment. Eleven patients were started on ART after initiation of MDR TB treatment and two declined ART. ART regimen data were available for 115 patients. The median duration of injectable use for the TB meds was 9.6 months (IQR 8.1–11.0 months)”.	“Extreme poverty was addressed by initiating monthly home visits and monthly patient visits to the treatment initiation site’s outpatient department, identification of a patient supporter to assist with DOT, psychosocial support, monthly food baskets, and social support for the most destitute patients. All patients in the program received food parcels to address the high rates of undernutrition. There is profound malnutrition and the gastrointestinal toxicities noted during MDR TB treatment in most cohorts from resource-constrained settings”.	Y
Mupere et al. (2012) [Low nutrient] [52]	Use of nutritional metrics such as BMI and weight to estimate food insecurity-related malnutrition, body wasting of participants using body mass index (BMI), and height-normalized indices (adjusted for height) for body composition of lean mass index (LMI) and fat mass index (FMI) [50]. Bioelectrical impedance analyzer (BIA) measures. Twenty-four-hour nutrient intake.	“There were no differences in average 24-h nutrient intake by Tuberculosis disease and HIV status”.	“There were no differences in average 24-h nutrient intake by Tuberculosis disease and HIV status. Loss of appetite, vomiting, fainting, loss of body mass, wasting, death”.	NR	NR	NA
Mupere et al. (2012) [Lean tissue] [53]	“Nutritional status was assessed using baseline height and weight anthropometric measurements and BIA before initiation of tuberculosis therapy. Lean tissue mass was calculated from BIA measurements using equations that were previously cross-validated in a sample of patients with and without HIV infection and have been applied elsewhere in African studies. Fat mass was calculated as body weight minus fat-free mass. Baseline wasting was defined using BMI and height-normalized indices (adjusted for height) for lean tissue mass and fat mass as measured by BIA. BMI can be partitioned into height-normalized indices of lean tissue mass index (LMI) and fat mass index (FMI), i.e., BMI = LMI + FMI as previously reported using BMI cutoff for malnutrition < 18.5 kg/m^2^. The cutoffs for low LMI and FMI corresponding to a BMI < 18.5 kg/m were as follows: LMI < 16.7 (kg/m^2^) for men and <14.6 (kg/m^2^) for women with corresponding FMI < 1.8 (kg/m^2^) for men and <3.9 (kg/m^2^) for women. LMI and FMI have the advantage of compensating for differences in height and age”.	Higher hazard of death among HIV seropositive participants.	“Loss of appetite, vomiting, fainting, loss of body mass, wasting, death”.	NR	NR	NA
Rudolph et al. (2013) [54]	“Nearly all adults (97% of females and 96% of males) reported being food insecure. Food insecurity was also relatively high (57%) among children as reported by parents/guardians. Reported levels of food security did not change throughout the study [52]”. “Mean dietary diversity scores for adults were below the minimum target of 7 out of 12 (adult males 6.1; adult females 5.0; child males 7.3; child females 7.5). Diets-particularly among adults-reflected a strong reliance on starchy staples (refined maize porridge and bread), with lower consumption of protein, fruit, and vegetables”. “The mean baseline BMI among adult males was low (19.2), while in adult females it was normal (23.3). All groups exhibited an increase in BMI, while waist to hip ratios remained stable. Child baseline median body weight was 18.1 kg for girls and 17.2 kg for boys, compared to WHO international growth standards of 18.2 kg for girls and 18.3 kg for boys”.	“67% of the adult TB patients (76% of females; 54% of males) self-reported as being HIV positive. There were no reports of HIV-positive serology in the child cohort. HIV negative adults showed greater improvement (mean change of 0.013, 95% CI −0.020–0.006) over three months than HIV positive adults (mean change of 0.007, 95% CI −0.021–0.082)”.	NR	NR	“The high rates of unemployment and self-reported HIV positive status among the adult cohort are above the national averages, but these findings are not surprising in the resource-poor setting of Alexandra and context of widespread co-infection with HIV and TB”.	Y
Sattler et al. (2018) [55]	“Of note, 7 (14%) participants had BMI < 16.5 kg/m^2^ and 17 (33%) had BMI 16.5–18.5 kg/m^2^”.	NR	“For the cohort of 51 participants, the 33 who survived were not different from the 18 who died”.	NR	NR	NA
Schact et al. (2019) [56]	A narrated description of food insecurity was used. The effect of treatment for HIV/AIDS is being hungry and this is a problem if no food at home.	NR	NR	Social-level barriers: stigma. Individual-level barriers: lack of prioritization of treatment, participants rapidly felt better and did not feel like completing the therapy, side effects of ART use such as pain in legs and articulations; itching; weakness and vertigo; swollen feet were mentioned, particularly in patients with TB/HIV co-infection [54]. Health facility-level barriers: delayed access to services, hygienic conditions were also mentioned as another negative aspect of the services, such as water cups used for patients to take their medications were not cleaned from one patient to the next.	NR	NA
Wang et al. (2020) [57]	Used the HFIAS. “Over half of all participants reported being food secure, and 15 (8.4%), 17 (9.5%) and 52 (29.0%) reported experiencing mild insecurity, moderate insecurity, and severe insecurity, respectively”.	NR	NR	“Among those who were HIV co-infected, the median duration since HIV diagnosis was 43 months, and 31(31.3%) had never taken ART, of whom 90% were newly diagnosed with HIV at or within one month of study enrolment ”.	NR	NA

NR—Not Reported; NA—Not Applicable.

## Data Availability

No new data were created or analyzed in this study.

## Supplementary Materials

The following supporting information can be downloaded at: https://www.mdpi.com/article/10.3390/ijerph19031101/s1, File S1: Consolidated Checklist for Reporting Scoping Reviews (Arksey-O’Malley framework and PRISMA Checklist).

## Appendix A

### PubMed Full Search Strategy

(Sub-Saharan Africa OR Sub-Saharan Africa OR Africa OR Central Africa OR Eastern Africa OR Northern Africa OR Southern Africa OR Western Africa OR Cameroon OR Central African Republic OR Chad OR Congo OR Equatorial Guinea OR Gabon OR Sao OR Principe OR Burundi OR Djibouti OR Eritrea OR Ethiopia OR Kenya OR Rwanda OR Somalia OR South Sudan OR Tanzania OR Uganda OR Angola OR Botswana OR Eswatini OR Lesotho OR Malawi OR Mozambique OR Namibia OR South Africa OR Zambia OR Zimbabwe OR Benin OR Burkina Faso OR Cabo Verde OR Cote d’Ivoire OR Ivory Coast OR Gambia OR Ghana OR Guinea OR Guinea-Bissau OR Liberia OR Mali OR Mauritania OR Niger OR Nigeria OR Senegal OR Sierra Leone OR Togo OR Algeria OR Egypt OR Libya OR Morocco OR Tunisia) AND (food security OR food insecurity OR food supply OR nutrition OR famine OR food desserts) AND (HIV infections OR acquired immunodeficiency syndrome OR HIV OR AIDS) AND (tuberculosis OR TB OR Koch disease OR Koch’s Disease).
